# Entwicklung der stationären Versorgungsqualität operativ behandelter Patienten mit einer proximalen Femurfraktur in Nordrhein-Westfalen: Eine Analyse über 61.249 Behandlungsverläufe auf Grundlage der Daten der externen stationären Qualitätssicherung

**DOI:** 10.1007/s00113-021-01065-9

**Published:** 2021-07-30

**Authors:** C. J. Neumann, U. Schulze-Raestrup, C. M. Müller-Mai, R. Smektala

**Affiliations:** 1 Klinik für Unfallchirurgie und Orthopädie, Knappschaftskrankenhaus Bochum-Langendreer, Universitätsklinikum der Ruhr-Universität Bochum, In der Schornau 23–25, 44892 Bochum, Deutschland; 2 Qualitätssicherung NRW, Ärztekammer Westfalen-Lippe, Münster, Deutschland; 3 Klinik für Unfallchirurgie, Orthopädie und Sportmedizin, Klinikum Lünen, Lünen, Deutschland

**Keywords:** Oberschenkelhalsfraktur, Pertrochantäre Femurfraktur, Alterstraumatologie, Versorgungsforschung, Registerdaten, Femoral neck fracture, Pertrochanteric femoral fracture, Geriatric traumatology, Care research, Register data

## Abstract

**Hintergrund und Fragestellung:**

Pro Jahr erleiden in Deutschland über 100.000 Menschen, überwiegend höheren Alters, eine proximale Femurfraktur. Steigende Fallzahlen im Zusammenhang mit einer alternden Bevölkerung und eine relativ große Zahl behandlungsbedürftiger Begleiterkrankungen erschweren die alltägliche Versorgung. Daher ist die Beobachtung der Versorgungsqualität dieser Patienten anhand relevanter Qualitätsparameter von großer Bedeutung, um Implikationen für die alltägliche Behandlungspraxis ableiten zu können.

**Material und Methoden:**

Die Daten der externen stationären Qualitätssicherung aus Nordrhein-Westfalen der Jahre 2007 und 2008 sowie 2017 und 2018 wurden analysiert und die Zeiträume vergleichend gegenübergestellt. Zusätzlich wurde anhand der dokumentierten Nebendiagnosen und weiterer Einflussparameter eine Risikoadjustierung mithilfe eines logistischen Regressionsmodells in Bezug auf die Zielgrößen der allgemeinen und chirurgischen Komplikationen sowie der Letalität durchgeführt. Es wurden sowohl osteosynthetisch als auch endoprothetisch versorgte Patienten berücksichtigt. Insgesamt konnten 61.249 Fälle in die Studie eingeschlossen werden.

**Ergebnisse:**

Positive Entwicklungen konnten im Bereich der chirurgischen Komplikationen und der Wundinfektionen mit Rückgängen um 1,2 % bzw. 0,8 % beobachtet werden. Bei Vorliegen einer Herz-Kreislauf-Erkrankung war das Outcome der Patienten besonders schlecht. Hierbei zeigten sich für die allgemeinen Komplikationen im Verlauf Verbesserungen in der Subkategorie der kardiovaskulären Ereignisse. Die Letalität lag unverändert bei 6 %. Deutlich gesteigert wurde die operative Tätigkeit an den Wochenenden. Patienten, deren Krankenhausaufnahme in zeitlichem Bezug zum Wochenende lag, wiesen kein erhöhtes Komplikations- oder Letalitätsrisiko auf. Obwohl der Anteil der erst nach über 48 h operierten Patienten von 11,4 % auf 8,2 % gesenkt werden konnte, verzögert sich die Operation (> 24 h) immer noch in 26,8 % der Fälle.

**Schlussfolgerungen:**

Vor dem Hintergrund steigender Leistungsanforderungen an das Gesundheitssystem dokumentieren die Ergebnisse Verbesserungen in einigen zentralen Bereichen der stationären Behandlung. Dennoch ist die Entwicklung von Strategien zur weiteren Reduktion der präoperativen Liegezeiten in medizinisch vertretbarer Weise zu fordern. Internistische Begleiterkrankungen beeinflussen das Outcome der Patienten maßgeblich. Somit ist eine adäquate Behandlung des multimorbiden Patientenkollektivs auf der Grundlage einer engen Kooperation zwischen alterstraumatologischen und geriatrisch-internistischen Fachabteilungen in der Alltagspraxis zu etablieren.

## Abstract (en)

**Background and objective:**

Every year in Germany over 100,000 people, mostly of older age, suffer a proximal femoral fracture. Increasing case numbers in connection with an aging population and a relatively large number of concomitant diseases requiring treatment make everyday care more difficult. It is therefore of great importance to observe the quality of care for these patients using relevant quality parameters in order to be able to derive implications for everyday treatment practice.

**Material and methods:**

The data of the external inpatient quality assurance from North Rhine-Westphalia for the years 2007–2008 and 2017–2018 were analyzed and the time periods compared. In addition, based on the documented secondary diagnosis and other influencing parameters, a risk adjustment was carried out with the aid of a logistic regression model with respect to the outcomes of general and surgical complications and mortality. Both osteosynthetically and endoprosthetically treated patients were taken into account. A total of 61,249 cases were included in the study.

**Results:**

Positive developments could be observed in the area of surgical complications and wound infections with decreases of 1.2% and 0.8%, respectively. Patients with cardiovascular diseases had a particularly poor outcome. Here, improvements in the subcategory of cardiovascular events were found for general complications. The mortality remained unchanged at 6%. Operative activity on the weekends increased significantly. Patients whose hospital admission was related to the weekend did not show an increased risk of complications or mortality. Although the proportion of patients who were operated on after more than 48 h was reduced from 11.4% to 8.2%, the operation (> 24 h) was still delayed in 26.8% of cases.

**Conclusion:**

Against the background of increasing performance demands on the healthcare system, the results document improvements in some central areas of inpatient treatment. Nevertheless, the development of strategies for the further reduction of the preoperative waiting times in a medically justifiable manner is required. Internal concomitant diseases have a decisive influence on patient outcome. Thus, an adequate treatment of the multimorbid patient collective is to be established in everyday practice on the basis of close cooperation between geriatric traumatology and geriatric internal medicine departments.

## Hintergrund

Jährlich werden in Deutschland über 100.000 Patienten mit einer proximalen Femurfraktur stationär versorgt [2]. Es handelt sich um eine der häufigsten verletzungsbedingten Diagnosen des älteren Patienten, die mit funktionellen Einbußen und einer erheblichen Einschränkung der Lebensqualität einhergehen kann [17]. In westlichen Ländern müssen zwischen 10 und 20 % der Betroffenen nach der akut-stationären Behandlung in einer Pflegeeinrichtung untergebracht werden [11]*.* Etwa ein Drittel der Patienten verstirbt postoperativ noch innerhalb des ersten Jahres [28]*.* Dabei ist anzunehmen, dass steigende Fallzahlen aufgrund der alternden Bevölkerung [33] zusammen mit einer hohen Zahl und Schwere relevanter Begleiterkrankungen im entsprechenden Patientengut zu einer stetigen Leistungsverdichtung beitragen und das Gesundheitssystem zunehmend stark beanspruchen. Somit ist die Beobachtung der diesbezüglichen Versorgungsqualität von immenser Bedeutung zur Klärung, ob dennoch ein adäquates Maß an Patientensicherheit gewährleistet werden kann.

Das Ziel dieser Untersuchung besteht darin, die Entwicklung der Versorgungssituation eines geriatrietypischen Kollektivs über die zurückliegende Dekade hinaus anhand relevanter Qualitätsindikatoren zu dokumentieren, um daraus Implikationen für die alltägliche Behandlungspraxis ableiten zu können. Insbesondere beim Qualitätsindikator der „präoperativen Verweildauer“ wurden in der Vergangenheit Mängel beobachtet [16], obwohl gezeigt werden konnte, dass Patienten in aller Regel von einer zeitnahen Operation binnen 24 h profitieren [9] und auch die deutschen Leitlinien die Einhaltung dieses Zeitfensters empfehlen [5, 10]. Vor diesem Hintergrund bekräftigte erst kürzlich der Gemeinsame Bundesausschuss (G-BA) in einer neuen Richtlinie zur Versorgung der hüftgelenknahen Femurfraktur die Forderung nach einer operativen Versorgung innerhalb 24 h, sofern der Allgemeinzustand des Patienten dies zulässt [12].

## Material und Methoden

### Datengrundlage und Datenverarbeitung

Im Rahmen der Sekundärdatennutzung wurden die von den Kliniken in Nordrhein-Westfalen dokumentierten Behandlungsverläufe der Patienten ab 65 Jahren mit einer hüftgelenknahen Femurfraktur ausgewertet. Hierzu wurde auf die Daten der externen stationären Qualitätssicherung NRW zurückgegriffen, und die Zeiträume der Jahre 2007 und 2008 sowie 2017 und 2018 wurden gegenübergestellt. Dabei erfolgte kein Vergleich einzelner Kliniken oder Klinikstandorte. Anhand der ICD-Codes (International Statistical Classification of Diseases and Related Health Problems) sowie der Angabe zur Frakturlokalisation wurde sichergestellt, dass nur Schenkelhals- und pertrochantäre Frakturen in die Untersuchung eingingen. Eine Identifikation der subtrochantären Frakturen war nur anhand der dokumentierten ICD-Codes möglich, da diese in den Erhebungsbögen nicht separat beschrieben, sondern ausschließlich unter der Kategorie „sonstige“ erfasst wurden. Um ein einheitliches Kollektiv mit einer präzisen und verlässlichen Angabe über die Frakturlokalisation zu erhalten, erfolgte ein Ausschluss subtrochantärer Frakturen. Berücksichtigt wurden auf Grundlage der dokumentierten Operationen- und Prozedurenschlüssel (OPS) weiterhin sowohl die osteosynthetisch als auch endoprothetisch versorgten Patienten. Solche Fälle, in denen kein Operationsverfahren verschlüsselt war, wurden aus den Betrachtungen ausgeschlossen. Somit wurde sichergestellt, dass jedem Behandlungsverlauf eindeutig ein primäres Operationsverfahren zugeordnet werden konnte. Die beobachteten Unterschiede wurden mithilfe des χ^2^-Tests und des t‑Tests auf statistische Signifikanz überprüft. Die Auswertung und die Erstellung von Abbildungen erfolgten mithilfe des Statistikprogramms SPSS 23 und Microsoft Excel 2016.

Im Zuge der Auswertung bestand Klarheit darüber, dass bei mehrfachem Testen derselben Grundgesamtheit ein multiples Testproblem besteht. Dies wurde bei einem angestrebten α‑Fehler von 0,05 entsprechend mathematisch berücksichtigt. Da das verwendete Programm SPSS den *p*-Wert nur bis auf 3 Nachkommastellen ausgibt, dieser jedoch nicht negativ sein kann, wurden Ergebnisse, bei denen *p* < 0,000 war, konservativ mit *p* < 0,001 angesetzt.

### Einschlusskriterien

Für die Untersuchung wurden Patienten ab 65 bis einschließlich 100 Jahren mit einer eindeutigen Geschlechtszuordnung und Angabe über die genaue Frakturlokalisation („medial“, „lateral“ oder „pertrochantär“) berücksichtigt. Weiterhin wurden nur solche Fälle eingeschlossen, bei denen eine klare Abgrenzung der Diagnose anhand des ICD-Codes möglich war, sodass Doppelnennungen oder fehlende Angaben des ICD-Codes zum Ausschluss führten. Für die Schenkelhalsfrakturen (SHF) wurden die Codes S72.0, S72.00, S72.01, S72.02, S72.03, S72.04 und S72.05 eingeschlossen. Pertrochantäre Frakturen (PTF) konnten mit S72.1, S72.10 sowie S72.11 einbezogen werden. Anhand der den Erhebungsbögen beigefügten Fachabteilungsschlüssel wurden nur diejenigen Patienten berücksichtigt, deren akut-stationäre Aufnahme über die Abteilungen Orthopädie, Unfallchirurgie oder allgemeine Chirurgie erfolgte. Die folgenden in den Erhebungsinstrumenten definierten Fachabteilungsschlüssel konnten mithin berücksichtigt werden: Orthopädie (2300, 2309, 2315, 2316), Unfallchirurgie (1600, 1690, 1691, 1692), allgemeine Chirurgie (1500, 1516, 1518, 1520, 1523, 1550, 1590, 1591, 1592). Ein Vergleich der Operationsverfahren zum historischen Zeitraum war ausschließlich mithilfe der OPS-Codes möglich. Diese wurden bis einschließlich zur 7. Stelle berücksichtigt und mussten entsprechend den im Zeitraum 2007 und 2008 erfassten Operationsverfahren umcodiert werden (Tab. 1)*.*

**Table 1:** 

OPS-Schlüssel	Umkodierung
5‑790.8; 5‑793.5; 5‑794.4	→ 1 → „DHS, Winkelplatte“
5‑790.3; 5‑790.4; 5‑790.5	→ 2 → „intramedulläre Stabilisierung“
5‑790.0	→ 3 → „Verschraubung“
5‑820.0	→ 4 → „Totalendoprothese“
5‑820.3	→ 5 → „monopolare Femurkopfprothese“
5‑820.4	→ 6 → „Duokopfprothese“

*DHS* Dynamische Hüftschraube

### Operationalisierung des logistischen Regressionsmodells

Im Weiteren wurde mithilfe eines multivariablen binären logistischen Regressionsmodells eine Risikoadjustierung anhand relevanter Einflussparameter in Bezug auf die Zielgrößen allgemeine Komplikationen^1^, spezifisch-chirurgische Komplikationen^2^ und Letalität durchgeführt. Es wurde ein binäres logistisches Regressionsmodell verwendet, da die Zielvariablen in den Datensätzen nicht skalar, sondern binär (ja/nein) kodiert sind [31]*.* Hierzu wurde auf ein etabliertes Datenmodell zurückgegriffen und entsprechend der Fragestellung angepasst [21, 23]*. *Zur Auswahl der unabhängigen Variablen wurde keine Forward oder Backward Selection durchgeführt. Basierend auf der Einschlussmethode gingen alle bereits evaluierten Einflussgrößen gleichzeitig in das Modell ein und wurden jeweils in Bezug auf einen der Ergebnisparameter (allgemeine Komplikationen, chirurgische Komplikationen oder Letalität) in gleicher Weise getestet. Als unabhängige Variablen wurden das Alter, das Geschlecht, die Frakturart, der Aufnahmewochentag, die präoperative Verweildauer, die Operationsverfahren, die Operationsdauer sowie die beiden Beobachtungszeiträume eingeschlossen. Zusätzlich wurden die von Bottle und Aylin [6] überprüften Nebendiagnosen berücksichtigt. Zu diesem Zweck wurden nach der Herangehensweise von Müller-Mai et al. [21] die einzelnen während des stationären Aufenthalts anhand der ICD-Codes dokumentierten Nebendiagnosen abgefragt und einer der Erkrankungskategorien aus Tab. 2 zugeordnet. Die Patienten wurden einer der Risikogruppen zugewiesen, sobald mindestens eine der Diagnosen vorlag. Die dem Modell zugrunde gelegten Werte beziehen sich auf den Zustand am Entlassungstag [21]*. *Als Risikomaß wurde die jeweilige „odds ratio“ (OR) berechnet. Die Modellgüte wurde mithilfe des Hosmer-Lemeshow-Tests überprüft.

**Table 2:** 

Erkrankungsgruppe	ICD-Code	Bedeutung
Karzinom	C	Alle Erkrankungen, deren ICD-Code mit einem C beginnt; Kapitel 2/bösartige Neubildungen (C00–C97)
Alzheimer/Demenz	F00	Demenz bei Alzheimer-Krankheit
	F01	Vaskuläre Demenz
	F02	Demenz bei andernorts klassifizierten Krankheiten
	F03	Nicht näher bezeichnete Demenz
	G30	Alzheimer-Krankheit
Diabetes mellitus	E10	Primär insulinabhängiger Diabetes mellitus (Typ-1-Diabetes)
	E11	Nicht primär insulinabhängiger Diabetes mellitus (Typ-2-Diabetes)
	E12	Diabetes mellitus in Verbindung mit Fehl- oder Mangelernährung (Malnutrition)
	E13	Sonstiger näher bezeichneter Diabetes mellitus
	E14	Nicht näher bezeichneter Diabetes mellitus
Koronare Herzerkrankung	I20	Angina pectoris
	I23	Bestimmte akute Komplikationen nach akutem Myokardinfarkt
	I24	Sonstige akute ischämische Herzkrankheit
	I25	Chronische ischämische Herzkrankheit
Chronische Atemwegserkrankung	J40	Bronchitis, nicht als akut oder chronisch bezeichnet
	J41	Einfache und schleimig-eitrige chronische Bronchitis
	J42	Nicht näher bezeichnete Bronchitis
	J43	Emphysem
	J44	Sonstige chronische obstruktive Lungenkrankheit
	J45	Asthma bronchiale
	J46	Status asthmaticus
Herzinsuffizienz	I50	Herzinsuffizienz
Hypertonus	I11	Hypertensive Herzkrankheit
	I12	Hypertensive Nierenkrankheit
	I13	Hypertensive Herz- und Nierenkrankheit
	I15	Sekundäre Hypertonie
Niereninsuffizienz	N17	Akutes Nierenversagen
	N18	Chronische Niereninsuffizienz
	N19	Nicht näher bezeichnete Niereninsuffizienz

Nach Müller-Mai et al. [21]

## Ergebnisse

### Darstellung der Grundgesamtheit

Insgesamt konnten unter Berücksichtigung der Ein- und Ausschlusskriterien die stationären Behandlungsverläufe von 61.249 Patienten aus Nordrhein-Westfalen einbezogen werden (Tab. 3). Während in den Jahren 2007 und 2008 noch 29.490 Patienten des Kollektivs der operativ-chirurgischen Versorgung zugeführt wurden, waren es im zweiten Studienzeitraum bereits 31.759 Patienten. Hieraus ergibt sich eine Fallzahlsteigerung um 7,7 %. Der Altersdurchschnitt lag insgesamt bei 82,5 Jahren. Dabei waren die Patienten der Jahre 2017 und 2018 im Schnitt um 7 Monate älter (82,8 Jahre) als noch im damaligen Zeitraum (82,2 Jahre). Von der Verletzung waren überwiegend Frauen betroffen. Das Geschlechterverhältnis lag bei 2,7:1 (w:m). Schenkelhalsfrakturen (SHF) wurden mit 67,5 % am häufigsten beobachtet. Mediale SHF waren mit 64,3 % weit häufiger vertreten als laterale SHF (3,3 %). Pertrochantäre Frakturen wurden in etwa einem Drittel der Fälle (32,5 %) diagnostiziert.

**Table 3:** 

Jahr der Erfassung	2007	2008	2017	2018	Gesamt
Anzahl der Patienten	14.550	14.940	16.090	15.669	61.249
Prozent (%)	23,8	24,4	26,3	25,6	100,0
Kumulierte Prozente	23,8	48,1	74,4	100,0	–

### Vergleich der Studienzeiträume

#### Wochentagsprofile.

**Figure 1:**
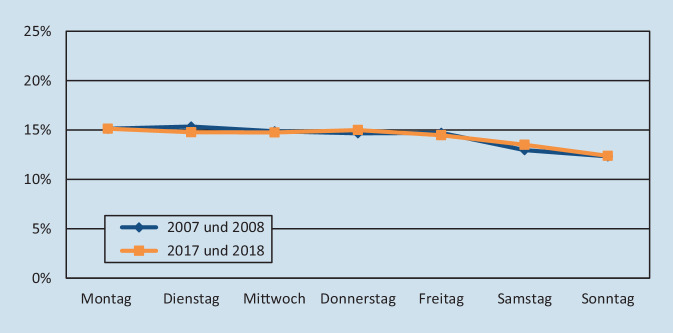

Bei der Aufnahmetätigkeit zeigten sich keine signifikanten Veränderungen im Vergleich der Beobachtungszeiträume (Abb. 1). Die meisten Patienten wurden im ersten Zeitraum dienstags (15,4 %) und im zweiten Abschnitt montags (15,1 %) aufgenommen. Die wenigsten Aufnahmen erfolgten jeweils am Sonntag (12,3 bzw. 12,4 %).

**Figure 2:**
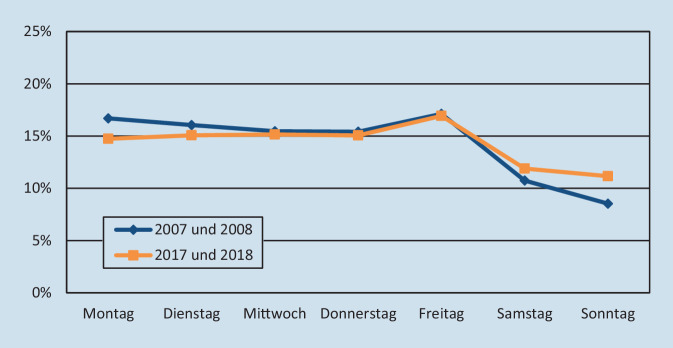

Deutliche Veränderungen konnten bei der operativen Tätigkeit beobachtet werden (Abb. 2). Die meisten Operationen fanden in beiden Abschnitten an einem Freitag statt (ca. 17 %). An den Wochenendtagen hat die operative Versorgung hin zum aktuelleren Zeitraum deutlich zugenommen. Der Anteil der operierten Patienten konnte am Samstag von 10,7 % auf 11,9 % und am Sonntag sogar von 8,5 % auf 11,2 % gesteigert werden, wobei sich die Veränderungen nach dem χ^2^-Test als statistisch signifikant darstellen (*p* < 0,001).

#### Nebendiagnosen.

Insgesamt wurden am häufigsten demenzielle Erkrankungen (17,5 %), Niereninsuffizienz (13,6 %), Diabetes mellitus (13,4 %) und Herzinsuffizienz (11,0 %) beschrieben. Chronische Atemwegserkrankungen und Bluthochdruck lagen mit 5,9 % bzw. 4,7 % im mittleren Häufigkeitsspektrum. Malignome (2,7 %) und ischämische Herzerkrankungen (1,1 %) wurden am wenigsten beobachtet. Signifikante Steigerungen der Dokumentationshäufigkeit zeigten sich nach dem χ^2^-Test für die Niereninsuffizienz, Alzheimer/Demenz, Diabetes mellitus, chronische Atemwegserkrankungen und Bluthochdruck.

#### Präoperative Verweildauer.

In den Jahren 2017 und 2018 wurde mit 73,2 % der überwiegende Anteil der Patienten innerhalb der ersten 24 h nach der Krankenhausaufnahme operiert (2007 und 2008: 72,8 %), wobei sich die Veränderungen zwischen den Zeiträumen als statistisch nicht signifikant erweisen (χ^2^-Test, *p* = 0,2547). Der Anteil der Patienten, der im Zeitintervall von 24–48 h operiert werden konnte, wurde von 15,8 % auf 18,6 % im letzten Zeitraum gesteigert (χ^2^-Test, *p* < 0,001). Weniger Patienten mussten in den Jahren 2017 und 2018 länger als 48 h auf ihre operative Versorgung warten (8,2 %), verglichen mit dem vorherigen Studienzeitraum (11,4 %). Der Unterschied ist signifikant (χ^2^-Test, *p* < 0,001).

#### Postoperative Liegezeiten.

Im ersten Zeitraum belief sich die durchschnittliche postoperative Liegezeit auf 16,33 Tage, im zweiten Zeitraum noch auf 14,17 Tage. Dies entspricht einem Rückgang um 2,16 Tage, der sich nach dem *t*-Test als statistisch signifikant darstellt (*p* < 0,001).

#### Operationsverfahren.

**Figure 3:**
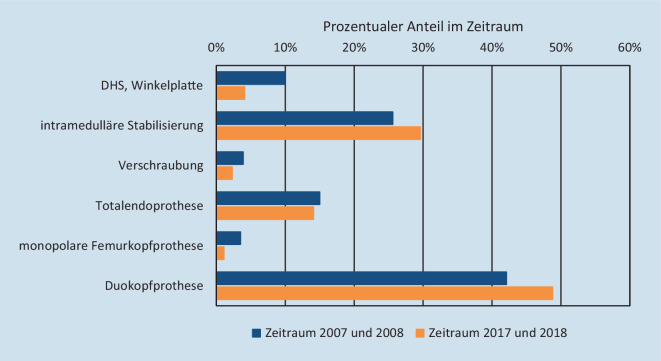

Insgesamt wurden 62,4 % des Patientenkollektivs primär mit einer Prothese versorgt. (Abb. 3). Dabei erfährt die Duokopfprothese eine deutliche Favorisierung. Ihr Einsatz hat sich von 42,1 % auf 48,8 % signifikant gesteigert (χ^2^-Test, *p* < 0,001). Unter den Osteosyntheseverfahren wird klar die intramedulläre Stabilisierung bevorzugt. Auch diese hat eine Zunahme (25,6 % gegenüber 29,6 %) erfahren (χ^2^-Test, *p* < 0,001). Die Anteile aller weiterer Verfahren waren rückläufig.

#### Operationsdauer.

Fasst man für die Auswertung alle operativen Eingriffe zusammen, so hat sich die durchschnittliche Operationszeit (Schnitt-Naht-Zeit) insgesamt von 66,72 auf 65,56 min reduziert. Tab. 4 bildet die eingeschlossenen Operationsverfahren und die dazugehörigen Operationszeiten in Minuten ab*.*

**Table 4:** 

			Dauer des Eingriffs (min)	
Zeitraum	Operationsverfahren	Anzahl	Mittelwert	Standardabweichung
2007 und 2008	DHS, Winkelplatte	2916	58,03	± 22,54
	Intramedulläre Stabilisierung	7547	53,09	± 20,68
	Verschraubung	1140	45,69	± 18,12
	TEP	4438	87,21	± 28,63
	Monopolare Femurkopfprothese	1027	66,75	± 21,21
	Duokopfprothese	12.422	71,65	± 23,29
2017 und 2018	DHS, Winkelplatte	1313	57,55	± 27,34
	Intramedulläre Stabilisierung	9396	44,56	± 20,37
	Verschraubung	726	44,34	± 19,74
	TEP	4471	84,26	± 32,36
	Monopolare Femurkopfprothese	356	58,03	± 22,54
	Duokopfprothese	15.497	53,09	± 20,68

*DHS* Dynamische Hüftschraube, *TEP* Totalendoprothese

#### Allgemeine Komplikationen.

Bei den allgemeinen Komplikationen konnten keine Veränderungen hinsichtlich des Anteils betroffener Patienten festgestellt werden (ca. 13,7 %). Hingegen konnten deutliche Unterschiede bei einzelnen Komplikationsarten (Tab. 5) beobachtet werden. Pneumonien wurden deutlich häufiger dokumentiert (2007/2008: 2,7 %; 2017/2018: 3,6 %). Auf der anderen Seite wurden kardiovaskuläre Komplikationen seltener verzeichnet als im Vorzeitraum (Rückgang von 5,3 % auf 3,9 %). Die Unterschiede sind dabei jeweils signifikant. Keine relevanten Veränderungen wurden bei den Thrombosen und Lungenembolien gesehen.

**Table 5:** 

		Zeitraum der Erfassung		χ^2^-Test
		2007 und 2008	2017 und 2018	
Pneumonie	Anzahl der Fälle	803	1132	*p* < 0,001
	% im Zeitraum	2,7	3,6	
Kardiovaskuläre Komplikation	Anzahl der Fälle	1.562	1253	*p* < 0,001
	% im Zeitraum	5,3	3,9	
Thrombose	Anzahl der Fälle	59	41	*p* = 0,030
	% im Zeitraum	0,2	0,1	
Lungenembolie	Anzahl der Fälle	202	202	*p* = 0,455
	% im Zeitraum	0,7	0,6	

#### Chirurgische Komplikationen.

Der Anteil von einer spezifisch-chirurgischen Komplikation betroffener Patienten konnte von 4,8 % auf 3,6 % reduziert werden (χ^2^-Test, *p* < 0,001). Differenziert man nach den einzelnen Komplikationsarten (Tab. 6), dann zeigen sich signifikante Rückgänge bei den Implantatfehllagen/-dislokationen und am deutlichsten bei den Blutungskomplikationen. Der Anteil der Kategorie „Wundhämatom/Nachblutung“ konnte von 2,1 % im damaligen Zeitraum auf nunmehr 1,0 % gesenkt werden.

**Table 6:** 

		Zeitraum der Erfassung		χ^2^-Test
		2007 und 2008	2017 und 2018	
Implantatfehllage	Anzahl der Fälle	54	31	*p* = 0,005
	% im Zeitraum	0,2	0,1	
Implantatdislokation	Anzahl der Fälle	152	106	*p* < 0,001
	% im Zeitraum	0,5	0,3	
Wundhämatom/ Nachblutung	Anzahl der Fälle	629	319	*p* < 0,001
	% im Zeitraum	2,1	1,0	
Gefäßläsion	Anzahl der Fälle	10	12	*p* = 0,800
	% im Zeitraum	0,0	0,0	
Nervenschaden	Anzahl der Fälle	27	27	*p* = 0,785
	% im Zeitraum	0,1	0,1	
Fraktur	Anzahl der Fälle	84	12	*p* < 0,001
	% im Zeitraum	0,3	0,0	

#### Letalität.

Für das Ereignis „Tod“ während des akut-stationären Aufenthalts konnten keine signifikanten Veränderungen zwischen den Zeiträumen beobachtet werden (χ^2^-Test, *p* = 0,726). Es verstarben rund 6 % der Patienten noch im Krankenhaus.

#### Infektionen.

**Figure 4:**
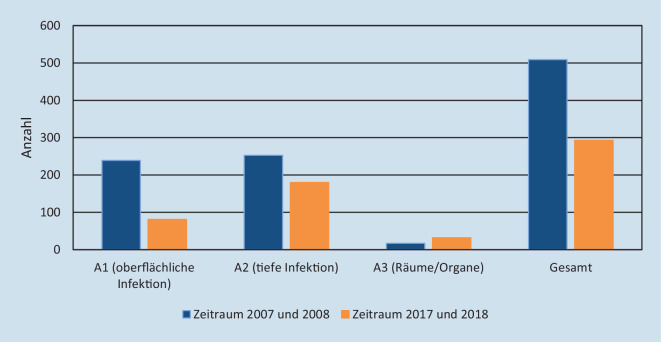

Insgesamt waren 802 Patienten (1,3 %) von einer postoperativen Wundinfektion betroffen (Abb. 4). Dabei wurde der Anteil von 1,7 % im damaligen Zeitraum auf 0,9 % in den jüngeren Jahren signifikant gesenkt (χ^2^-Test, *p* < 0,001). Diese positive Entwicklung ergibt sich aus den Veränderungen bei den oberflächlichen und tiefen Infektionen. Schwere Infektionen von Organen und Körperhöhlen haben bei insgesamt sehr geringen Anteilen zugenommen (0,06 % gegenüber 0,1 %).

### Logistische Regression

Insgesamt wurden drei binäre logistische Regressionsanalysen in Bezug auf die Endpunkte Letalität, chirurgische Komplikationen und allgemeine Komplikationen durchgeführt. Dabei war jedes Modell statistisch signifikant (χ^2^-Test, *p* < 0,001). Der Hosmer-Lemeshow-Test erwies sich für die allgemeinen und die chirurgischen Komplikationen bei einem angestrebten Signifikanzniveau von 5 % als nicht signifikant. Es besteht also kein Anlass, die Modellgültigkeit für diese Endpunkte anzuzweifeln. Hinsichtlich der Letalität wurde das Ziel einer geeigneten Anpassungsgüte nicht erreicht. Der Hosmer-Lemeshow-Test war hier signifikant. Dennoch sollen der Vollständigkeit halber auch diese Ergebnisse berichtet werden, um Einflusstendenzen der Risikofaktoren ableiten zu können.

#### Allgemeine Komplikationen.

Die Variable „Alter“ war mit einer leichten Risikoerhöhung für allgemeine postoperative Komplikationen assoziiert (Tab. 7). Deutlicher war der Einfluss des Geschlechts. Frauen hatten ein 27,5 % niedrigeres Risiko. Für den Wochentag der Aufnahme war kein Einfluss nachweisbar. Bei Vorliegen einer PTF war das Risiko um 25 % gesteigert. Bezüglich der Nebendiagnosen konnte für die ischämischen Herzerkrankungen eine 5‑fache Risikoerhöhung berechnet werden. Die Herz- und Niereninsuffizienz sowie Atemwegserkrankungen zeigten ebenfalls ein signifikant erhöhtes Risiko.

**Table 7:** 

		Allgemeine Komplikationen	Chirurgische Komplikationen	Letalität
		OR [95 %-KI]	OR [95 %-KI]	OR [95 %-KI]
Alter		1,024 [1,021; 1,028]	1,005 [0,999; 1,010]	1,058 [1,052; 1,064]
Geschlecht	Männlich	Referenzkategorie	Referenzkategorie	Referenzkategorie
	Weiblich	0,725 [0,688; 0,764]	1,148 [1,045; 1,262]	0,585 [0,542; 0,631]
Frakturart	MSHF	Referenzkategorie	Referenzkategorie	Referenzkategorie
	LSHF	1,060 [0,919; 1,222]	1,123 [0,897; 1,406]	1,062 [0,863; 1,307]
	PTF	1,250 [1,091; 1,432]	1,568 [1,275; 1,930]	1,072 [0,869; 1,323]
Wochentag der Aufnahme	Montag	Referenzkategorie	Referenzkategorie	Referenzkategorie
	Dienstag	0,942 [0,863; 1,027]	0,987 [0,852; 1,145]	0,944 [0,832; 1,071]
	Mittwoch	0,972 [0,891; 1,061]	1,117 [0,967; 1,290]	0,913 [0,804; 1,038]
	Donnerstag	0,987 [0,905; 1,077]	1,040 [0,898; 1,205]	0,923 [0,812; 1,048]
	Freitag	0,975 [0,893; 1,064]	1,019 [0,878; 1,182]	0,914 [0,803; 1,039]
	Samstag	1,009 [0,924; 1,103]	1,085 [0,934; 1,261]	0,871 [0,762; 0,995]
	Sonntag	1,011 [0,923; 1,107]	1,088 [0,934; 1,268]	1,045 [0,916; 1,191]
Präoperative Verweildauer	< 24 h	Referenzkategorie	Referenzkategorie	Referenzkategorie
	24–48 h	1,172 [1,101; 1,248]	0,992 [0,892; 1,104]	1,176 [1,074; 1,288]
	> 48 h	1,301 [1,207; 1,403]	1,054 [0,928; 1,198]	1,288 [1,157; 1,433]
Malignom	Nein	Referenzkategorie	Referenzkategorie	Referenzkategorie
	Ja	1,035 [0,902; 1,187]	0,821 [0,630; 1,068]	1,828 [1,550; 2,156]
Demenz	Nein	Referenzkategorie	Referenzkategorie	Referenzkategorie
	Ja	0,919 [0,864; 0,977]	0,898 [0,804; 1,004]	0,944 [0,865; 1,031]
Diabetes mellitus	Nein	Referenzkategorie	Referenzkategorie	Referenzkategorie
	Ja	0,934 [0,871; 1,001]	0,890 [0,787; 1,006]	0,947 [0,855; 1,048]
IHE	Nein	Referenzkategorie	Referenzkategorie	Referenzkategorie
	Ja	5,020 [4,290; 5,873]	0,990 [0,687; 1,427]	3,235 [2,647; 3,955]
CTAE	Nein	Referenzkategorie	Referenzkategorie	Referenzkategorie
	Ja	1,190 [1,086; 1,305]	0,932 [0,781; 1,112]	1,217 [1,070; 1,384]
Herzinsuffizienz	Nein	Referenzkategorie	Referenzkategorie	Referenzkategorie
	Ja	1,527 [1,428; 1,634]	1,189 [1,049; 1,346]	1,764 [1,613; 1,929]
Hypertension	Nein	Referenzkategorie	Referenzkategorie	Referenzkategorie
	Ja	0,951 [0,856; 1,056]	0,979 [0,810; 1,183]	0,951 [0,823; 1,099]
Nierenversagen	Nein	Referenzkategorie	Referenzkategorie	Referenzkategorie
	Ja	1,500 [1,407; 1,598]	1,108 [0,982; 1,250]	1,520 [1,392; 1,661]
Operationsverfahren	DHS/W	Referenzkategorie	Referenzkategorie	Referenzkategorie
	IMS	1,055 [0,943; 1,180]	0,858 [0,707; 1,042]	1,271 [1,064; 1,519]
	VS	0,749 [0,597; 0,939]	1,844 [1,330; 2,556]	0,763 [0,530; 1,099]
	TEP	1,155 [0,990; 1,348]	1,831 [1,432; 2,342]	1,399 [1,094; 1,788]
	FKP	1,488 [1,219; 1,817]	1,604 [1,145; 2,247]	1,627 [1,201; 2,203]
	DKP	1,291 [1,119; 1,490]	1,599 [1,260; 2,029]	1,562 [1,246; 1,957]
Operationsdauer		1,003 [1,002; 1,003]	1,011 [1,010; 1,013]	1,000 [0,998; 1,001]
Zeitraum	2007 und 2008	Referenzkategorie	Referenzkategorie	Referenzkategorie
	2017 und 2018	0,954 [0,907; 1,003]	0,745 [0,686; 0,810]	0,906 [0,841; 0,976]

*MSHF* mediale Schenkelhalsfraktur, *LSHF* laterale Schenkelhalsfraktur, *PTF* pertrochantäre Fraktur, *W* Winkelplatte, *IMS* intramedulläre Stabilisierung, *VS* Verschraubung, *FKP* Femurkopfprothese, *DKP* Duokopfprothese, *IHE* ischämische Herzerkrankung, *CTAE* chronische Atemwegserkrankung

#### Chirurgische Komplikationen.

Für das Alter und den Aufnahmewochentag wurde kein risikoerhöhender Einfluss beobachtet. Frauen hatten ein um 14,8 % höheres Risiko für eine chirurgische Komplikation. Bei den pertrochantären Frakturen bestand ein um 57 % erhöhtes Risiko, verglichen mit medialen SHF. Unter den Nebendiagnosen war einzig die Herzinsuffizienz mit einer signifikanten Steigerung des Risikos verbunden.

#### Letalität.

Das Alter zeigte einen geringfügigen Einfluss auf die Letalität. Frauen hatten, verglichen mit Männern, ein um 41,5 % niedrigeres Risiko, während der stationären Behandlung zu versterben. Für die Frakturlokalisation, den Aufnahmewochentag und die Operationsdauer konnte keine Risikoerhöhung nachgewiesen werden. Der Samstag als Aufnahmetag war sogar mit einer geringfügig niedrigeren Letalität verbunden*.* Unter den Operationsverfahren bestand die stärkste Risikoerhöhung bei Implantation einer Femurkopfprothese, verglichen mit der Kategorie DHS/Winkelplatte. Hinsichtlich der Begleiterkrankungen zeigte sich die deutlichste Risikoerhöhung bei Vorliegen einer ischämischen Herzerkrankung (3-fach erhöhtes Risiko), bei Malignomerkrankungen und Herzinsuffizienz (Risikosteigerung um jeweils ca. 80 %).

#### Präoperative Verweildauer.

**Figure 5:**
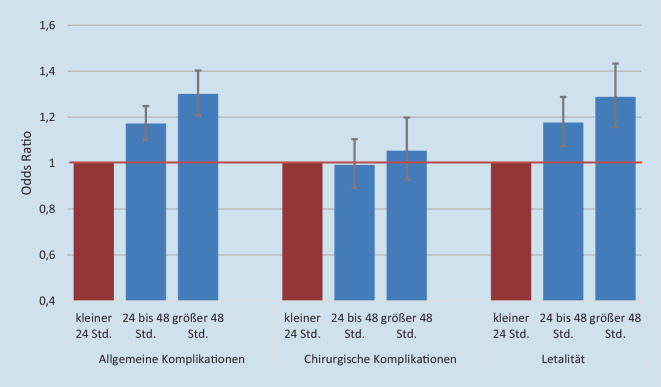

Als Referenzkategorie wurde der Zeitraum „kleiner 24 h“ definiert. Mathematisch wird dabei eine Odds Ratio von 1,0 zugrunde gelegt, und die Zeitintervalle von „24–48 h“ und „größer 48 h“ werden mit dieser Referenzkategorie verglichen (Abb. 5).

Dabei zeigte sich kein Einfluss der Liegezeiten auf die chirurgischen Komplikationen. Hingegen konnten signifikante Einflüsse in ähnlich starker Ausprägung für die allgemeinen Komplikationen und die Letalität nachgewiesen werden. Mussten Patienten zwischen 24 und 48 h bis zu ihrer operativen Versorgung warten, dann war ihr Risiko um ca. 17 % erhöht. Bei einer präoperativen Liegezeit oberhalb 48 h lag die Risikoerhöhung bei rund 30 %.

## Diskussion

Vor dem Hintergrund der demografischen Entwicklung und steigender Leistungsanforderungen an die stationäre Versorgung ist die Auswertung der dargestellten Parameter von besonderem Interesse. Aus den gewonnenen Informationen lassen sich einerseits durch die Definition konkreter Qualitätsziele Konsequenzen für die alltägliche und praktische Versorgung älterer Patienten mit einer proximalen Femurfraktur ableiten. Andererseits liefert die Untersuchung Evidenz für die Wirksamkeit und Effektivität der bislang vollzogenen Maßnahmen und bietet Argumente für den wissenschaftlichen und gesundheitspolitischen Diskurs [25].

Die Auswertung der Behandlungsdaten zeigte im Ergebnis einen Rückgang der chirurgischen Komplikationen, eine unveränderte Rate allgemeiner Komplikationen und eine unveränderte Letalitätsrate. Wundinfektionen wurden seltener beschrieben. Die Operationstätigkeit an den Wochenenden konnte gesteigert werden, und die Rate verzögert operierter Patienten sank im Behandlungsverlauf.

Im Zuge des Erhebungsverfahrens wurde bei jedem Dokumentationsfall zur Erfassung der Komplikationen zunächst erfragt, ob eine behandlungsbedürftige allgemeine postoperative bzw. spezifisch-chirurgische Komplikation vorlag (Antwortoptionen: ja/nein). Berücksichtigt man allein diese Zählung, so ergaben sich positive Entwicklungen bei den chirurgischen Komplikationen, während für die allgemeinen Komplikationen keine Veränderungen beobachtet wurden. In einem weiteren Schritt wurde dann eine Spezifizierung der Art der vorliegenden Komplikation verlangt. Verschlechterungen konnten für keine dieser Kategorien aus spezifisch-chirurgischer Sicht festgestellt werden. Es konnten sogar deutliche Verbesserungen im Bereich der Blutungskomplikationen und der Implantatfehllagen/-dislokationen gesehen werden. Bei den allgemeinen Komplikationen zeigten sich die deutlichsten Verbesserungen bei den kardiovaskulären Ereignissen, was ein Hinweis darauf sein könnte, dass diagnostische und therapeutische Möglichkeiten im Zuge der stationären Behandlung besser genutzt bzw. das intra- und postoperative Monitoring verbessert werden konnten. Zu beachten ist, dass sich aufgrund der durchschnittlich kürzeren stationären Verweildauer im zweiten Studienabschnitt (2007 und 2008 postoperativ: 16,33 Tage; 2017 und 2018: 14,17 Tage) damit auch der zeitliche Rahmen für das Auftreten von Komplikationen als verkürzt darstellt [32]*. *Da diese nur für die Zeit der stationären Behandlung dokumentiert werden konnten, erscheinen die Ergebnisse im Vergleich der Zeiträume ggf. günstiger. Pneumonien wurden hingegen insgesamt häufiger beschrieben. Die Veränderung der Pneumonieraten zeigt eine Entwicklung auf, die zur Ursachen- und Erklärungssuche veranlassen sollte. Möglicherweise trägt die steigende Resistenzentwicklung zu einer erschwerten Therapie bereits prästationär bei, sodass nichtausgeheilte Pneumonien häufiger bereits aus dem Pflegeheim oder der häuslichen Umgebung mit ins Krankenhaus gebracht werden. Letztlich kann nicht mit Sicherheit gesagt werden, ob eine Komplikation, wie beispielsweise eine Pneumonie, nicht bereits zum Aufnahmezeitpunkt vorgelegen hat und somit keine Komplikation im Rahmen der Behandlung darstellt. Deshalb sind die Früherkennung und Prophylaxe der Komplikationen von immenser Bedeutung. Zum anderen könnte gerade auch im stationären Setting eine steigende Antibiotikaresistenz zu dieser Tendenz beitragen. Besonders eine wachsende Zahl nosokomialer Infektionen und die Verbreitung multiresistenter Bakterien mit hoher Pathogenität [37] bieten somit einen relevanten Erklärungsansatz. Auf der anderen Seite führt ggf. auch die sich verändernde Altersstruktur zusammen mit einer zunehmenden Zahl und Schwere von Begleiterkrankungen sowie steigender Immobilität im Alter zu dieser Entwicklung.

Hinsichtlich der Delirraten kann mangels expliziter Angaben in den Behandlungsdaten und einer vermutlich hohen Zahl nichtdiagnostizierter oder erfasster Fälle im stationären Verlauf keine verlässliche Aussage getroffen werden. Tatsächlich stellt dieser Aspekt bei der postoperativen Nachbehandlung eine zentrale Information dar, und es wäre wünschenswert, die Ausbildung eines Delirs im Sinne einer postoperativen Komplikation mitabzubilden. Es ist zu hoffen, dass gerade auch vor diesem Hintergrund der dringend notwendige Ansatz des orthogeriatrischen Komanagements zu einer Verbesserung hinsichtlich der Delirerkennung, -behandlung und epidemiologischen Abbildung beitragen kann.

Bezüglich der Formulierung einzelner Items in den Fragebögen bestehen Unterschiede zwischen den Zeiträumen. So wurde für die Auswertung z. B. das Item „Implantatfehllage“ (2007 und 2008) mit dem Item „primäre Implantatfehllage“ (2017 und 2018) verglichen. Insgesamt jedoch scheinen diese Unterschiede im Vergleich zu früheren Arbeiten [32] abgenommen zu haben. Jedem Item aus dem damaligen Zeitraum konnte nun ein konkretes Item aus dem aktuelleren Zeitraum gegenübergestellt werden, das den Anschein erweckt, ein und denselben Sachverhalt abzubilden, sodass die Vergleichbarkeit als gegeben anzunehmen ist.

Die Erfassung der postoperativen Wundinfektionen hat sich im Beobachtungszeitraum nicht verändert und wurde nach der Definition der Centers for Disease Control and Prevention (CDC) vorgenommen, sodass eine direkte Gegenüberstellung zulässig erscheint. Bei insgesamt sehr geringen Infektionsraten konnten hier deutliche Verbesserungen beobachtet werden.

Rund 6 % der Patienten verstarben während der stationären Behandlung, was sich mit den Angaben in der Literatur deckt [14, 21]. Es ist davon auszugehen, dass bei steigenden Leistungsanforderungen und erhöhtem Patientenaufkommen gewisse Anpassungsprozesse erforderlich waren, um die Sterblichkeit zumindest auf einem konstanten Niveau zu halten. Die verwendeten Operationsverfahren zeigten deutliche Unterschiede bezüglich der Letalität. Am höchsten war der Anstieg der Letalitätsrate unter Verwendung einer monopolaren Femurkopfprothese (OR: 1,63), was nicht überrascht, da diese heutzutage allenfalls zur Schmerzbehandlung und zur Herstellung der Lagerungsfähigkeit bei Patienten mit niedriger Lebenserwartung eingesetzt werden sollte [4]*. *Die Totalendoprothese (TEP) weist niedrigere Revisionsraten und bessere funktionelle Ergebnisse auf als die Hemiendoprothese, zeigt dabei aber höhere Dislokationsrisiken, eine größere Invasivität mit höheren Blutverlusten und längerer Operationsdauer [15, 36]*. *Sie stellt somit eine Behandlungsoption für Patienten mit hohem Aktivitäts- und Anspruchsgrad dar [4]*.* Die Dynamische Hüftschraube (DHS) bietet trotz der Gefahr höherer Revisionsraten bei entsprechender Knochensubstanz auch bei älteren Patienten eine probate Behandlungsoption und vermeidet gleichzeitig die Komplikationen der deutlich invasiveren Endoprothetik [19, 39]*.* Obwohl es sich insgesamt um ein geriatrietypisches Kollektiv handelt, ist grundsätzlich zu erwarten, dass dabei biologisch jüngere Patienten eher mit einem weniger invasiven hüftkopferhaltenden Verfahren operiert wurden, was zu einer gewissen Verzerrung der Ergebnisse führen könnte (z. B. niedrigere allgemeine Komplikationsraten unter Verschraubung).

Hinsichtlich der Operationsverfahren zeigte sich für das ältere Patientenkollektiv insgesamt eine klare Steigerung der endoprothetischen Versorgung in Form der Duokopfprothese, womit der Trend früherer Jahre fortgesetzt wurde [32]*.* Die Bevorzugung der Duokopfprothese erscheint medizinisch konsequent, da gezeigt werden konnte, dass ältere Patienten nach Versorgung mit einer Hemiendoprothese im Verlauf weniger Schmerzen, eine höhere Patientenzufriedenheit und eine höhere Lebensqualität aufwiesen als solche, die einem Osteosyntheseverfahren unterzogen wurden [13]. Allerdings werden PTF bevorzugt osteosynthetisch versorgt [29]*.* Die eigenen Daten belegen in diesem Zusammenhang höhere Komplikationsraten bei Patienten mit einer PTF im Vergleich zur medialen SHF (25 % höhere Rate allgemeiner und 75 % gesteigerte Rate chirurgischer Komplikationen). Im Rahmen einer Metaanalyse wurden ebenfalls geringere Komplikationsraten und bessere gesundheitliche Outcomes für ältere Patienten unter endoprothetischer Versorgung jeglicher Art im Vergleich zu intramedullärer Fixierung gesehen [15]*.* Bei einem Großteil der älteren Patientenklientel kann eine Osteoporose als wesentliche Mitursache der Fraktur nachgewiesen werden [35]*.* Da Patienten mit einer PTF im Schnitt älter sind als solche mit einer SHF [22]*,* ist anzunehmen, dass hier u. a. die Problematik einer schwächeren Knochensubstanz insgesamt zu einer erschwerten operativen Versorgung beiträgt und sich dieser Effekt auch auf den Anstieg der allgemeinen Komplikationen aufgrund verzögerter Remobilisierung im weiteren Verlauf überträgt.

Mit Blick auf die benötigten Operationszeiten (Schnitt-Naht-Zeiten) scheint ebenfalls eine gewisse Professionalisierung stattgefunden zu haben. Im zweiten Zeitraum wurden die Patienten schneller operiert und hatten dabei gleichzeitig weniger chirurgische Komplikationen.

Im Rahmen einer bundesweiten Studie für die Jahre 2004 und 2008 konnte gezeigt werden, dass die Operation von Patienten, deren Aufnahme auf einen Freitag oder Samstag fiel, deutlich häufiger erst nach über 48 h erfolgte [31]. In der Konsequenz wurde eine Verbesserung der Krankenhausorganisation sowie der personellen Ausstattung gefordert. Die eigene Auswertung zeigte nun, dass die Zahl der am Wochenende operierten Patienten signifikant gesteigert werden konnte, was darauf schließen lässt, dass vermehrt personelle und strukturelle Gründe beseitigt wurden, die bislang einer Operation am Wochenende entgegenstanden. Patienten, die kurz vor dem Wochenende oder am Wochenende selbst aufgenommen wurden, wiesen kein erhöhtes Risiko hinsichtlich der Komplikationen oder der Letalität auf. Dennoch liegt die Operationstätigkeit am Wochenende immer noch deutlich unter der Aufnahmetätigkeit und auch unter der Operationstätigkeit der Wochentage.

Der Anteil der Patienten mit einer präoperativen Liegedauer oberhalb 48 h wurde signifikant um 3,2 % reduziert. Wichtig ist diese Beobachtung, da zahlreiche Autoren eine deutliche Zunahme der Letalität nach verzögerter Operation feststellten [6, 21, 30]. Ebenfalls anhand von Registerdaten konnte gezeigt werden, dass eine vorbestehende Antikoagulation maßgeblich zu einer Verzögerung des Operationszeitpunktes beiträgt [18]. In diesem Zusammenhang ist wiederum die Bedeutung der geriatrisch-internistischen Mitbetreuung zum Zwecke eines professionalisierten Gerinnungsmanagements hervorzuheben.

Mit einem Maximum der Verletzungszahlen um das 87. bzw. 88. Lebensjahr handelt es sich um ein hochbetagtes und – wie anhand der dokumentierten Begleiterkrankungen nachzuvollziehen ist – multimorbides Patientenkollektiv. Eine besonders schlechte Prognose hatten Patienten bei Vorliegen einer ischämischen Herzkrankheit bzw. einer Herz- oder Niereninsuffizienz. Dabei überrascht es nicht, dass eine begleitende geriatrische Mitbehandlung nicht nur zu besseren Ergebnissen im Rahmen der Rehabilitation [3], sondern auch zu einer signifikanten Reduktion der peri- und postoperativen Komplikationen führen kann [24]. Bereits Buecking et al. [8] sahen auf der Grundlage einer durchgeführten Metaanalyse deutliche Hinweise in der Literatur, dass eine frühzeitig begonnene interdisziplinäre Kooperation zwischen unfallchirurgisch-geriatrischen Fachabteilungen zu einer Verbesserung des Behandlungsergebnisses alterstraumatologischer Patienten führen kann. Um die spezifischen, häufig geriatrisch-internistischen, Anforderungen von multimorbiden Patienten mit Fragilitätsfrakturen besser berücksichtigen zu können, wurden Versorgungsmodelle im Sinne eines orthogeriatrischen Komanagements entwickelt. Entsprechende Studien zum Outcome der in dieser Weise behandelten Patienten zeigten vielversprechende Ergebnisse. Eine randomisierte kontrollierte Studie aus Norwegen sah bei frühzeitiger Zusammenarbeit eine höhere Mobilität der Patienten 4 Monate nach dem Frakturereignis [26]. Rapp et al. [27] fanden, dass die adjustierte 30-Tages-Mortalität um 22 % niedriger war, sofern die Behandlung in einem Krankenhaus mit orthogeriatrischem Komanagement durchgeführt wurde. Im Ausbau des interdisziplinären Versorgungsansatzes scheint auch der Schlüssel zur Reduktion der Komplikationen während der stationären Behandlung zu liegen. Um eine Steigerung der interdisziplinären Behandlungsqualität zu erreichen, wurde durch die Deutsche Gesellschaft für Unfallchirurgie das Zertifizierungsverfahren AltersTraumaZentrum DGU® initiiert [7]*.* Seit dem Start im Jahr 2014 wurden bereits 108 Zentren in Deutschland, Österreich und der Schweiz zertifiziert [1]. Durch die weitere Einführung des AltersTraumaRegister DGU®, an dem alle Alterstraumazentren teilnehmen müssen, sollen Daten für die alterstraumatologische Versorgungsforschung gewonnen und dem internationalen Vergleich zugänglich gemacht werden [7]*.*

Die Zusammenschau der Ergebnisse dieser Untersuchung dokumentiert zunächst die gestiegenen Anforderungen an das deutsche Gesundheitssystem im Zusammenhang mit einer älteren Patientenklientel und liefert Hinweise auf eine verbesserte Versorgungsqualität proximaler Femurfrakturen in Nordrhein-Westfalen. Gleichwohl ist immer noch ein klares Verbesserungspotenzial im Rahmen der Behandlung festzustellen.

## Limitationen

Die beobachteten Veränderungen der Versorgungsqualität erfordern eine sehr sorgfältige Bewertung sowie eine Überprüfung der Datenvalidität und ihrer Einschränkungen. So beziehen sich die zur Verfügung stehenden Dokumentationsdaten ausschließlich auf die Zeit der stationären Behandlung. Auf diese Weise werden jedoch die Auswirkungen des Frakturereignisses im Nachgang der Krankenhausbehandlung, wie z. B. das Auftreten poststationärer Komplikationen, ein reduziertes Maß selbstständiger Lebensführung und eine möglicherweise neu entstandene Pflegebedürftigkeit nicht erfasst. Auch die hohe Einjahresmortalität von rund 30 % [21, 28] wird hierbei systematisch unterschätzt, sodass die weitergehende Abbildung der Patientenverläufe nur unter Einbeziehung zusätzlicher Quellen, wie z. B. Krankenkassendaten, möglich ist. Insgesamt können durch die verwendeten Registerdaten eine hohe Fallzahl und statistische Power generiert, allerdings aufgrund des Studiendesigns keine Aussagen über Kausalzusammenhänge getroffen werden [18]*.* Durch den Vergleich der gewählten Beobachtungszeiträume können auch Langzeitverläufe betrachtet werden, jedoch ist damit keine zeitlich kontinuierliche Darstellung des gesamten Zeitintervalls der Jahre 2007–2018 gewährleistet und etwaige zwischenzeitliche Schwankungen der dargestellten Parameter können nicht ausgeschlossen werden.

Das Dokumentationsverfahren unterlag starken Veränderungen hinsichtlich der Erhebungsinstrumente sowie der Anzahl und Formulierung einzelner Items. Zudem wurden ab 2015 osteosynthetisch und endoprothetisch behandelte Patienten in jeweils eigenen Modulen erfasst. Es ist unklar, welchen Einfluss diese Veränderungen auf die Dokumentationspraxis hatten. Die Vergleichbarkeit der Zeiträume könnte hierdurch z. T. eingeschränkt sein. Weiterhin ist unklar, inwieweit beobachtete Veränderungen durch eine strengere Dokumentationspraxis der Anwender zu erklären sind, die sich möglicherweise über die Jahre hinweg etabliert hat. Zudem können Eingabefehler des dokumentierenden Personals nicht ausgeschlossen werden [18]*,* zumal ein gewisser Interpretationsspielraum zwischen den realen Gesundheitsproblemen des Patienten und den verfügbaren ICD- und OPS-Codes besteht [23]*.* Dieser Spielraum könnte weiterhin zum Zwecke der Erlösoptimierung im Sinne eines „right coding“ ausgenutzt worden sein [23], was zu einer Verzerrung des realen Bildes führen könnte [34]*.*

Der alleinige Vergleich einzelner Items zur Messung des abstrakten Qualitätsbegriffs erscheint indes nicht ausreichend, bedenkt man, dass sich patientenbezogene Faktoren, wie z. B. das Alter, das Geschlecht und Begleiterkrankungen [20]*,* mitunter stärker auf das Behandlungsergebnis auswirken als klinikbezogene Faktoren [38]*.* Aus diesem Grund wurde eine Risikoadjustierung vorgenommen. Dabei ist zu beachten, dass es sich um ein multivariables Modell handelt, bei dem alle unabhängigen Variablen gleichzeitig einfließen. Abhängigkeiten der Kovariaten untereinander können nicht ausgeschlossen werden. Darüber hinaus existieren Einflussvariablen, die nicht berücksichtigt werden können, wie z. B. der sozioökonomische Status. Dennoch lassen sich Tendenzen hinsichtlich des risikoerhöhenden bzw. -senkenden Effektes einzelner Einflussparameter in Bezug auf die getesteten Ergebnisparameter identifizieren.

## Fazit für die Praxis

- Anzahl und Altersdurchschnitt der versorgten Patienten sind klar gestiegen, bei unveränderter stationärer Letalität von 6 %.
- Weniger Patienten hatten eine chirurgische Komplikation. Am stärksten sind Blutungskomplikationen zurückgegangen. Verbesserungen zeigten sich auch bei den Wundinfektionen.
- Pneumonien wurden jüngst häufiger und kardiovaskuläre Ereignisse deutlich seltener beschrieben. Hier sollte eine strenge Dokumentation gepflegt und weiter beobachtet werden.
- Die Operationstätigkeit am Wochenende konnte gesteigert werden. Patienten haben kein erhöhtes Risiko, wenn sie kurz vor dem Wochenende oder am Wochenende selbst aufgenommen werden.
- Zu fordern ist eine gleichbleibende, frühzeitige und hochqualitative Versorgung proximaler Femurfrakturen an 7 Tagen/Woche.
- Strategien zur Reduktion der präoperativen Liegezeiten in medizinisch vertretbarer Weise müssen entwickelt und diskutiert werden.
- Es ist eine klare Bevorzugung der endoprothetischen Versorgung der SHF in Form der Duokopfprothese festzustellen.
- Eine enge Kooperation zwischen alterstraumatologischen und geriatrisch-internistischen Fachabteilungen ist zu etablieren.

## Footnotes

1 Allgemeine behandlungsbedürftige postoperative Komplikation(en): Pneumonie, kardiovaskuläre Komplikation(en), tiefe Bein‑/Beckenvenenthrombose, Lungenembolie, Sonstige.

2 Behandlungsbedürftige intra-/postoperative chirurgische Komplikation(en): Implantatfehllage, Implantatdislokation, Wundhämatom/Nachblutung, Gefäßläsion, Nervenschaden, Fraktur, Sonstige.
